# Ethyl [(2-hydroxy­phen­yl)(pyridinium-2-ylamino)meth­yl]phospho­nate methanol solvate

**DOI:** 10.1107/S1600536808015675

**Published:** 2008-06-07

**Authors:** Ming-Xia Li, Miao-Li Zhu, Li-Ping Lu

**Affiliations:** a Institute of Molecular Science, Key Laboratory of Chemical Biology and Molecular Engineering of the Education Ministry, Shanxi University, Taiyuan, Shanxi 030006, People’s Republic of China

## Abstract

In the title compound, C_14_H_17_N_2_O_4_P·CH_3_OH, the planes of the pyridinium-2-ylamino and 2-hydroxy­phenyl groups form a dihedral angle of 75.6 (1)°, with the pyridinium NH group and the 2-hydroxy­phenyl OH group pointing in opposite directions. Three intra­molecular hydrogen bonds are observed. Two phospho­nate and two methanol mol­ecules are connected by O—H⋯O hydrogen bonds as a centrosymmetric dimeric cluster, and inter­act further with other dimeric clusters via N—H⋯O, O—H⋯O and C—H⋯O hydrogen bonds and C—H⋯π inter­actions, resulting in a sheet structure.

## Related literature

For related literature, see: Bernstein *et al.* (1995 ▶); Briceño *et al.* (2007 ▶); Foster & Weinhold (1980 ▶); Jeffrey *et al.* (1985 ▶); Kaboudin & Moradi (2005 ▶); Kachkovskyi & Kolodiazhnyi (2007 ▶); Kafarski & Lejczak (2001 ▶); Liu *et al.* (2002 ▶); Meyer *et al.* (2004 ▶); Palacios *et al.* (2005 ▶); Rohovec *et al.* (1999 ▶).
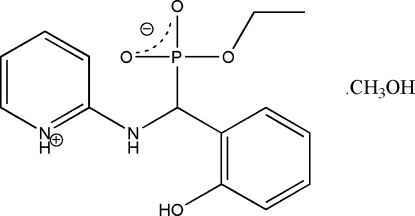


## Experimental

### 

#### Crystal data

C_14_H_17_N_2_O_4_P·CH_4_O
*M* *_r_* = 340.31
Monoclinic, 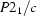

*a* = 12.821 (3) Å
*b* = 9.536 (2) Å
*c* = 16.567 (3) Å
β = 122.308 (14)°
*V* = 1711.9 (6) Å^3^
*Z* = 4
Mo *K*α radiation
μ = 0.19 mm^−1^
*T* = 298 (2) K
0.40 × 0.20 × 0.20 mm

#### Data collection

Bruker SMART 1K CCD diffractometer
Absorption correction: multi-scan (*SADABS*; Sheldrick, 2000 ▶) *T*_min_ = 0.875, *T*_max_ = 0.964
6784 measured reflections
2991 independent reflections
2419 reflections with *I* > 2σ(*I*)
*R*_int_ = 0.034

#### Refinement

*R*[*F* ^2^ > 2σ(*F* ^2^)] = 0.067
*wR*(*F* ^2^) = 0.147
*S* = 1.14
2991 reflections
210 parameters
1 restraint
H-atom parameters constrained
Δρ_max_ = 0.34 e Å^−3^
Δρ_min_ = −0.34 e Å^−3^

### 

Data collection: *SMART* (Bruker, 2000 ▶); cell refinement: *SAINT* (Bruker, 2000 ▶); data reduction: *SAINT*; program(s) used to solve structure: *SHELXS97* (Sheldrick, 2008 ▶); program(s) used to refine structure: *SHELXL97* (Sheldrick, 2008 ▶); molecular graphics: *SHELXTL/PC* (Sheldrick, 2008 ▶); software used to prepare material for publication: *PLATON* (Spek, 2003 ▶) and *publCIF* (Westrip, 2008 ▶).

## Supplementary Material

Crystal structure: contains datablocks I, global. DOI: 10.1107/S1600536808015675/cf2201sup1.cif

Structure factors: contains datablocks I. DOI: 10.1107/S1600536808015675/cf2201Isup2.hkl

Additional supplementary materials:  crystallographic information; 3D view; checkCIF report

## Figures and Tables

**Table 1:** Hydrogen-bond geometry (Å, °) *Cg* is the centroid of the C1–C6 ring.

*D*—H⋯*A*	*D*—H	H⋯*A*	*D*⋯*A*	*D*—H⋯*A*
N1—H1*A*⋯O3	0.87	2.57	2.957 (3)	108
N2—H2*A*⋯O3^i^	0.87	1.86	2.692 (3)	160
N1—H1*A*⋯O3^i^	0.87	2.03	2.813 (3)	150
O1—H1⋯O4^ii^	0.82	1.81	2.618 (3)	170
C7—H7⋯O1	0.98	2.28	2.783 (4)	111
C9—H9⋯O1	0.93	2.55	3.438 (5)	160
C12—H12⋯O5^i^	0.93	2.46	3.169 (5)	133
O5—H5*A*⋯O4	0.82	1.98	2.795 (4)	176
C14—H14*B*⋯*Cg*^iii^	0.96	2.91	3.697 (8)	141

Symmetry codes: (i) 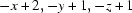; (ii) 
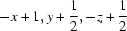
; (iii) 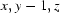.

**Fig. 1.**
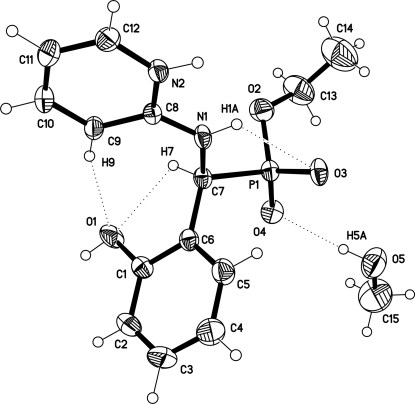
The molecular structure of (I) with displacement ellipsoids drawn at the 30% probability level. Dotted lines indicate intramolecular hydrogen bonds.

**Fig. 2.**
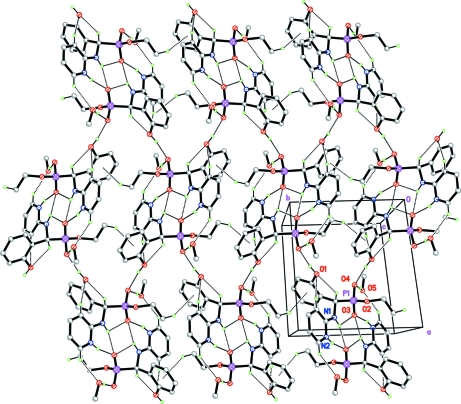
The dimer formed via R44(4), R12(6) and R33(9) rings, and the two-dimensional sheet formed through R66(34) rings. Dotted lines indicate hydrogen bonds and C—H···π interactions.

## supplementary crystallographic information

### Comment

Organophosphorus compounds are of importance because of their growing applications in medicine and agriculture. Aminophosphonates, one family of organophosphorus compounds, have received much attention as phosphorus analogs of naturally occurring aminocarboxylic acids. Many of these types of compounds have antibacterial, anticancer, and enzyme inhibitory properties, and so on (Kafarski & Lejczak, 2001; Liu *et al.*, 2002; Meyer *et al.*, 2004). Many new aminophosphonate compounds have been synthesized and characterized (Palacios *et al.*, 2005; Kaboudin & Moradi, 2005; Kachkovskyi & Kolodiazhnyi, 2007) for these reasons. The title compound was synthesized in order to understand its inhibitory activity on the protein tyrosine phosphatase 1B (PTP1B). Here we describe the crystal structure.

The planes of the pyridinium-2-amino and 2-hydroxyphenyl groups form a dihedral angle of 75.6 (1)°, with the N—H group of pyridinium and O—H of 2-hydroxyphenyl pointing in opposite directions. When the ethyl group and one of the two O atoms bonded to P are substituted by phenyl groups, the dihedral angle between the 2-hydroxyphenyl and pyridine rings is 54.9 (1)°, and the N atom of the pyridine ring and O—H of 2-hydroxyphenyl are close together, forming an intramolecular hydrogen bond (Rohovec *et al.*, 1999). Thus the substitution of functional groups around the P atom influences the arrangement of other function groups. The ethyl (2-hydroxyphenyl)(pyridinium-2-ylamino)methylphosphonate molecule displays three intramolecular hydrogen bonds, two C—H···O and one N—H···O. N1—H1···O3 and C7—H7···O1 lead to the formation of five-membered S(5) ring motifs (Bernstein *et al.*, 1995; Briceño *et al.*, 2007). C9—H9···O1 results in an eight-membered S(8) ring motif. Thus, O1 is involved in a bifurcated hydrogen bond (Jeffrey *et al.*, 1985), which produces a distorted seven-membered ring. Additionally, the solvent methanol is hydrogen bonded to O4, stabilizing the molecular conformation.

The intermolecular interactions of compound (I) are shown in Fig. 2 and in the hydrogen bonding table. Two ethyl (2-hydroxyphenyl)(pyridinium-2-ylamino)methylphosphonate molecules are connected antiparallel as a centrosymmetric dimer *via* bifurcated hydrogen bonds in which N1 and N2 are donors and O3 is the acceptor, giving rise to two hydrogen-bonded *R*^1^_2_(6) rings. In the bifurcated hydrogen bond, the two interactions are unequal; the N···O distance of 2.694 (3) Å and angle of 161° are obviously a stronger interaction than the N···O distance of 2.813 (3) Å and the angle of 150°. Two intermolecular N—H···O hydrogen bonds together with two intramolecular N—H···O interactions form another *R*^4^_4_(4) ring. The methanol molecules also link the dimers through O—H···O and C—H···O hydrogen bonds, generating two *R*^3^_3_(9) rings. Thus five hydrogen-bonded rings, namely two *R*^3^_3_(9), two *R*^1^_2_(6) and one *R*^4^_4_(4), form a complicated hydrogen-bonding network (Fig. 2). In this network, O3 acts as an acceptor of three hydrogen atoms and forms a trifurcated hydrogen bond (Jeffrey *et al.*, 1985), which is not observed very often. Meanwhile, O3 and one of its equivalents by symmetry share H1A, forming a bifurcated hydrogen bond. Neighbouring dimers are linked to each other *via* O—H···O hydrogen bonds. Four such dimers constitute a repeat unit with a thirty-four-membered *R*^6^_6_(34) ring, generating two-dimensional sheets parallel to (102). A C—H···π weak interaction involving ethyl and hydroxyphenyl groups also helps to stabilize the crystal structure (Fig. 2).

### Experimental

A solution of 1.882 g (0.02 mol) pyridin-2-amine in 20 ml of ethanol was added dropwise to a stirred solution of an equimolar amount of salicyaldehyde (0.02 mol, 3.1 ml) in 20 ml of ethanol and refluxed for 2 h. A solution of diethyl phosphonate (0.04 mol, 5.13 ml) in 10 ml of ethanol was then added dropwise. The mixture was refluxed for about 30 h until a solid appeared. The precipitate was collected and washed with ethanol and diethyl ether. A white solid was obtained (2.107 g, yield 34.4%). Colorless crystals were obtained from methanol.

### Refinement

The C13—C14 bond length was restrained to 1.50 (1) Å, because free refinement gave an unacceptably short bond, possibly due to unresolved disorder. H atoms attached to C atoms of (I) were placed in geometrically idealized positions with C*sp*^2^—H = 0.93, *Csp*^3^(methyl)—H = 0.96, and *Csp*^3^(methylene)—H = 0.97 Å and constrained to ride on their parent atoms, with *U*_iso_(H) = 1.2*U*_eq_(C) (1.5*U*_eq_for methyl H). H atoms attached to N and O atoms were located in a difference Fourier map and refined as riding, with *U*_iso_ = 1.2*U*_eq_(N,O).

### Crystal data

**Table:**

C_14_H_17_N_2_O_4_P·CH_4_O	*F*_000_ = 720
*M**_r_* = 340.31	*D*_x_ = 1.320 Mg m^−^^3^
Monoclinic, *P*2_1_/*c*	Mo *K*α radiation λ = 0.71073 Å
Hall symbol: -P 2ybc	Cell parameters from 1906 reflections
*a* = 12.821 (3) Å	θ = 2.5–23.8º
*b* = 9.536 (2) Å	µ = 0.19 mm^−^^1^
*c* = 16.567 (3) Å	*T* = 298 (2) K
β = 122.308 (14)º	Block, colourless
*V* = 1711.9 (6) Å^3^	0.40 × 0.20 × 0.20 mm
*Z* = 4	

### Data collection

**Table:**

Bruker SMART 1K CCD diffractometer	2991 independent reflections
Radiation source: fine-focus sealed tube	2419 reflections with *I* > 2σ(*I*)
Monochromator: graphite	*R*_int_ = 0.034
*T* = 298(2) K	θ_max_ = 25.0º
ω scans	θ_min_ = 1.9º
Absorption correction: multi-scan(SADABS; Sheldrick, 2000)	*h* = −15→15
*T*_min_ = 0.875, *T*_max_ = 0.964	*k* = −11→8
6784 measured reflections	*l* = −17→19

### Refinement

**Table:**

Refinement on *F*^2^	Secondary atom site location: difference Fourier map
Least-squares matrix: full	Hydrogen site location: inferred from neighbouring sites
*R*[*F*^2^ > 2σ(*F*^2^)] = 0.067	H-atom parameters constrained
*wR*(*F*^2^) = 0.147	*w* = 1/[σ^2^(*F*_o_^2^) + (0.052*P*)^2^ + 1.1778*P*] where *P* = (*F*_o_^2^ + 2*F*_c_^2^)/3
*S* = 1.14	(Δ/σ)_max_ = 0.001
2991 reflections	Δρ_max_ = 0.34 e Å^−^^3^
210 parameters	Δρ_min_ = −0.34 e Å^−^^3^
1 restraint	Extinction correction: none
Primary atom site location: structure-invariant direct methods	

### Special details

**Table:**

Geometry. All e.s.d.'s (except the e.s.d. in the dihedral angle between two l.s. planes) are estimated using the full covariance matrix. The cell e.s.d.'s are taken into account individually in the estimation of e.s.d.'s in distances, angles and torsion angles; correlations between e.s.d.'s in cell parameters are only used when they are defined by crystal symmetry. An approximate (isotropic) treatment of cell e.s.d.'s is used for estimating e.s.d.'s involving l.s. planes.
Refinement. Refinement of *F*^2^ against ALL reflections. The weighted *R*-factor *wR* and goodness of fit *S* are based on *F*^2^, conventional *R*-factors *R* are based on *F*, with *F* set to zero for negative *F*^2^. The threshold expression of *F*^2^ > σ(*F*^2^) is used only for calculating *R*-factors(gt) *etc*. and is not relevant to the choice of reflections for refinement. *R*-factors based on *F*^2^ are statistically about twice as large as those based on *F*, and *R*- factors based on ALL data will be even larger.

### Fractional atomic coordinates and isotropic or equivalent isotropic displacement parameters (Å^2^)

**Table:**

	*x*	*y*	*z*	*U*_iso_*/*U*_eq_	
P1	0.75798 (7)	0.45914 (9)	0.40161 (6)	0.0359 (3)	
O1	0.5572 (2)	0.7872 (3)	0.22842 (16)	0.0561 (7)	
H1	0.5007	0.8424	0.1964	0.067*	
O2	0.7741 (2)	0.3583 (3)	0.33273 (18)	0.0578 (7)	
O3	0.86639 (19)	0.4487 (2)	0.50109 (15)	0.0466 (6)	
O4	0.63500 (19)	0.4400 (2)	0.38884 (15)	0.0481 (6)	
N1	0.8906 (2)	0.6464 (3)	0.37455 (17)	0.0390 (7)	
H1A	0.9521	0.6023	0.4221	0.047*	
N2	1.0392 (2)	0.7331 (3)	0.35182 (17)	0.0381 (6)	
H2A	1.0852	0.6775	0.3996	0.046*	
C1	0.6172 (3)	0.8248 (3)	0.3218 (2)	0.0417 (8)	
C2	0.5805 (3)	0.9373 (4)	0.3538 (3)	0.0552 (10)	
H2	0.5115	0.9893	0.3105	0.066*	
C3	0.6448 (4)	0.9723 (4)	0.4481 (3)	0.0681 (12)	
H3	0.6190	1.0475	0.4690	0.082*	
C4	0.7468 (4)	0.8976 (5)	0.5121 (3)	0.0713 (12)	
H4	0.7905	0.9215	0.5765	0.086*	
C5	0.7848 (3)	0.7865 (4)	0.4808 (2)	0.0545 (10)	
H5	0.8549	0.7364	0.5246	0.065*	
C6	0.7210 (3)	0.7479 (3)	0.3857 (2)	0.0373 (7)	
C7	0.7639 (3)	0.6274 (3)	0.3523 (2)	0.0352 (7)	
H7	0.7091	0.6217	0.2827	0.042*	
C8	0.9195 (3)	0.7286 (3)	0.3241 (2)	0.0336 (7)	
C9	0.8361 (3)	0.8096 (3)	0.2451 (2)	0.0415 (8)	
H9	0.7530	0.8119	0.2249	0.050*	
C10	0.8774 (3)	0.8845 (4)	0.1985 (2)	0.0531 (10)	
H10	0.8219	0.9379	0.1457	0.064*	
C11	1.0008 (4)	0.8832 (4)	0.2277 (3)	0.0559 (10)	
H11	1.0282	0.9341	0.1946	0.067*	
C12	1.0803 (3)	0.8069 (4)	0.3050 (3)	0.0479 (9)	
H12	1.1637	0.8051	0.3260	0.057*	
C13	0.7568 (5)	0.2100 (5)	0.3340 (4)	0.0900 (15)	
H13A	0.7122	0.1760	0.2687	0.108*	
H13B	0.7055	0.1931	0.3598	0.108*	
C14	0.8694 (6)	0.1287 (6)	0.3889 (5)	0.126 (2)	
H14A	0.9171	0.1363	0.3600	0.189*	
H14B	0.8489	0.0322	0.3899	0.189*	
H14C	0.9165	0.1640	0.4530	0.189*	
O5	0.6628 (3)	0.3431 (4)	0.5586 (2)	0.0890 (10)	
H5A	0.6514	0.3728	0.5080	0.107*	
C15	0.5492 (6)	0.3157 (8)	0.5475 (5)	0.125 (2)	
H15A	0.4896	0.2940	0.4818	0.187*	
H15B	0.5226	0.3967	0.5661	0.187*	
H15C	0.5571	0.2375	0.5868	0.187*	

### Atomic displacement parameters (Å^2^)

**Table:**

	*U*^11^	*U*^22^	*U*^33^	*U*^12^	*U*^13^	*U*^23^
P1	0.0292 (4)	0.0369 (5)	0.0371 (5)	−0.0019 (4)	0.0146 (4)	0.0050 (4)
O1	0.0374 (13)	0.0611 (17)	0.0480 (15)	0.0195 (12)	0.0082 (11)	0.0025 (12)
O2	0.0709 (17)	0.0395 (14)	0.0736 (17)	−0.0025 (12)	0.0458 (15)	−0.0012 (12)
O3	0.0325 (12)	0.0521 (15)	0.0422 (13)	0.0002 (10)	0.0113 (10)	0.0174 (11)
O4	0.0320 (12)	0.0570 (15)	0.0480 (13)	−0.0098 (11)	0.0164 (11)	−0.0010 (11)
N1	0.0282 (13)	0.0438 (16)	0.0367 (14)	0.0043 (12)	0.0117 (12)	0.0146 (12)
N2	0.0345 (14)	0.0380 (15)	0.0376 (15)	0.0027 (12)	0.0165 (12)	0.0066 (12)
C1	0.0335 (17)	0.0407 (19)	0.048 (2)	−0.0008 (15)	0.0203 (16)	0.0013 (16)
C2	0.052 (2)	0.044 (2)	0.071 (3)	0.0119 (17)	0.034 (2)	0.0047 (19)
C3	0.087 (3)	0.051 (3)	0.077 (3)	0.009 (2)	0.050 (3)	−0.012 (2)
C4	0.094 (3)	0.060 (3)	0.053 (2)	−0.001 (2)	0.035 (2)	−0.013 (2)
C5	0.059 (2)	0.049 (2)	0.044 (2)	0.0051 (18)	0.0207 (18)	0.0018 (17)
C6	0.0353 (17)	0.0355 (18)	0.0393 (18)	−0.0013 (14)	0.0188 (15)	0.0040 (14)
C7	0.0259 (15)	0.0401 (18)	0.0308 (16)	0.0009 (13)	0.0093 (13)	0.0038 (13)
C8	0.0355 (17)	0.0302 (16)	0.0327 (16)	0.0000 (13)	0.0165 (14)	0.0021 (13)
C9	0.0400 (18)	0.045 (2)	0.0394 (18)	0.0051 (15)	0.0210 (15)	0.0112 (15)
C10	0.061 (2)	0.052 (2)	0.043 (2)	0.0107 (19)	0.0250 (18)	0.0176 (17)
C11	0.066 (3)	0.052 (2)	0.065 (2)	0.001 (2)	0.045 (2)	0.0142 (19)
C12	0.047 (2)	0.047 (2)	0.059 (2)	−0.0012 (17)	0.0350 (19)	0.0043 (18)
C13	0.100 (4)	0.048 (3)	0.121 (4)	−0.006 (3)	0.059 (3)	−0.009 (3)
C14	0.135 (5)	0.074 (4)	0.176 (6)	0.008 (4)	0.088 (5)	0.001 (4)
O5	0.073 (2)	0.127 (3)	0.0708 (19)	0.0061 (19)	0.0411 (17)	0.0241 (19)
C15	0.113 (5)	0.144 (6)	0.158 (6)	−0.003 (4)	0.099 (5)	0.024 (5)

### Geometric parameters (Å, °)

**Table:**

P1—O4	1.486 (2)	C5—H5	0.930
P1—O3	1.486 (2)	C6—C7	1.502 (4)
P1—O2	1.588 (3)	C7—H7	0.980
P1—C7	1.821 (3)	C8—C9	1.399 (4)
O1—C1	1.357 (4)	C9—C10	1.351 (4)
O1—H1	0.820	C9—H9	0.930
O2—C13	1.433 (5)	C10—C11	1.386 (5)
N1—C8	1.334 (4)	C10—H10	0.930
N1—C7	1.473 (4)	C11—C12	1.347 (5)
N1—H1A	0.869	C11—H11	0.930
N2—C8	1.347 (4)	C12—H12	0.930
N2—C12	1.347 (4)	C13—C14	1.451 (6)
N2—H2A	0.870	C13—H13A	0.970
C1—C2	1.385 (5)	C13—H13B	0.970
C1—C6	1.387 (4)	C14—H14A	0.960
C2—C3	1.363 (5)	C14—H14B	0.960
C2—H2	0.930	C14—H14C	0.960
C3—C4	1.364 (6)	O5—C15	1.391 (6)
C3—H3	0.930	O5—H5A	0.821
C4—C5	1.378 (5)	C15—H15A	0.960
C4—H4	0.930	C15—H15B	0.960
C5—C6	1.381 (4)	C15—H15C	0.960
			
O4—P1—O3	116.22 (13)	C6—C7—H7	107.7
O4—P1—O2	111.04 (14)	P1—C7—H7	107.7
O3—P1—O2	110.63 (14)	N1—C8—N2	116.9 (3)
O4—P1—C7	109.88 (14)	N1—C8—C9	125.5 (3)
O3—P1—C7	108.56 (13)	N2—C8—C9	117.6 (3)
O2—P1—C7	99.12 (14)	C10—C9—C8	119.3 (3)
C1—O1—H1	109.5	C10—C9—H9	120.4
C13—O2—P1	120.4 (3)	C8—C9—H9	120.4
C8—N1—C7	123.6 (2)	C9—C10—C11	121.3 (3)
C8—N1—H1A	115.6	C9—C10—H10	119.3
C7—N1—H1A	120.8	C11—C10—H10	119.3
C8—N2—C12	123.1 (3)	C12—C11—C10	118.6 (3)
C8—N2—H2A	112.8	C12—C11—H11	120.7
C12—N2—H2A	123.9	C10—C11—H11	120.7
O1—C1—C2	122.6 (3)	N2—C12—C11	120.0 (3)
O1—C1—C6	117.3 (3)	N2—C12—H12	120.0
C2—C1—C6	120.1 (3)	C11—C12—H12	120.0
C3—C2—C1	120.5 (4)	O2—C13—C14	115.2 (4)
C3—C2—H2	119.7	O2—C13—H13A	108.5
C1—C2—H2	119.7	C14—C13—H13A	108.5
C2—C3—C4	120.2 (4)	O2—C13—H13B	108.5
C2—C3—H3	119.9	C14—C13—H13B	108.5
C4—C3—H3	119.9	H13A—C13—H13B	107.5
C3—C4—C5	119.7 (4)	C13—C14—H14A	109.5
C3—C4—H4	120.2	C13—C14—H14B	109.5
C5—C4—H4	120.2	H14A—C14—H14B	109.5
C4—C5—C6	121.4 (4)	C13—C14—H14C	109.5
C4—C5—H5	119.3	H14A—C14—H14C	109.5
C6—C5—H5	119.3	H14B—C14—H14C	109.5
C5—C6—C1	118.1 (3)	C15—O5—H5A	109.1
C5—C6—C7	120.9 (3)	O5—C15—H15A	109.5
C1—C6—C7	120.9 (3)	O5—C15—H15B	109.5
N1—C7—C6	112.7 (3)	H15A—C15—H15B	109.5
N1—C7—P1	107.5 (2)	O5—C15—H15C	109.5
C6—C7—P1	113.4 (2)	H15A—C15—H15C	109.5
N1—C7—H7	107.7	H15B—C15—H15C	109.5
			
O4—P1—O2—C13	−55.0 (3)	C1—C6—C7—P1	−116.5 (3)
O3—P1—O2—C13	75.6 (3)	O4—P1—C7—N1	174.96 (19)
C7—P1—O2—C13	−170.5 (3)	O3—P1—C7—N1	46.9 (2)
O1—C1—C2—C3	−179.0 (3)	O2—P1—C7—N1	−68.6 (2)
C6—C1—C2—C3	−0.9 (5)	O4—P1—C7—C6	49.7 (2)
C1—C2—C3—C4	0.6 (6)	O3—P1—C7—C6	−78.4 (2)
C2—C3—C4—C5	0.1 (7)	O2—P1—C7—C6	166.1 (2)
C3—C4—C5—C6	−0.6 (6)	C7—N1—C8—N2	−178.6 (3)
C4—C5—C6—C1	0.3 (5)	C7—N1—C8—C9	1.3 (5)
C4—C5—C6—C7	179.4 (3)	C12—N2—C8—N1	177.1 (3)
O1—C1—C6—C5	178.6 (3)	C12—N2—C8—C9	−2.7 (5)
C2—C1—C6—C5	0.4 (5)	N1—C8—C9—C10	−177.7 (3)
O1—C1—C6—C7	−0.5 (4)	N2—C8—C9—C10	2.1 (5)
C2—C1—C6—C7	−178.7 (3)	C8—C9—C10—C11	−0.4 (5)
C8—N1—C7—C6	−78.5 (4)	C9—C10—C11—C12	−0.9 (6)
C8—N1—C7—P1	155.8 (2)	C8—N2—C12—C11	1.5 (5)
C5—C6—C7—N1	−58.0 (4)	C10—C11—C12—N2	0.4 (6)
C1—C6—C7—N1	121.0 (3)	P1—O2—C13—C14	−99.2 (5)
C5—C6—C7—P1	64.4 (4)		

### Hydrogen-bond geometry (Å, °)

**Table:**

*D*—H···*A*	*D*—H	H···*A*	*D*···*A*	*D*—H···*A*
N1—H1A···O3	0.87	2.57	2.957 (3)	108
N2—H2A···O3^i^	0.87	1.86	2.692 (3)	160
N1—H1A···O3^i^	0.87	2.03	2.813 (3)	150
O1—H1···O4^ii^	0.82	1.81	2.618 (3)	170
C7—H7···O1	0.98	2.28	2.783 (4)	111
C9—H9···O1	0.93	2.55	3.438 (5)	160
C12—H12···O5^i^	0.93	2.46	3.169 (5)	133
O5—H5A···O4	0.82	1.98	2.795 (4)	176
C14—H14B···Cg^iii^	0.96	2.91	3.697 (8)	141

Symmetry codes: (i) −*x*+2, −*y*+1, −*z*+1; (ii) −*x*+1, *y*+1/2, −*z*+1/2; (iii) *x*, *y*−1, *z*.
